# Causal relationship between physical activity, leisure sedentary behaviors and COVID-19 risk: a Mendelian randomization study

**DOI:** 10.1186/s12967-022-03407-6

**Published:** 2022-05-13

**Authors:** Xiong Chen, Xiaosi Hong, Wenjing Gao, Shulu Luo, Jiahao Cai, Guochang Liu, Yinong Huang

**Affiliations:** 1 Department of Pediatric Urology, Guangzhou Women and Children’s Medical Center, Guangzhou Medical University, Guangzhou, China; 2 Department of Pediatric Surgery, Guangzhou Institute of Paediatrics, Guangzhou Medical University, Guangzhou, Guangdong China; 3 Department of Endocrinology, Sun Yat-Sen Memorial Hospital, Sun Yat-Sen University, Guangzhou, China; 4 Clinical Data Center of Guangzhou Women and Children’s Medical Center, Guangzhou Medical University, Guangzhou, China; 5 Department of Prosthodontics, Hospital of Stomatology, Guangdong Provincial Key Laboratory of Stomatology, Guanghua School of Stomatology, Sun Yat-Sen University, Guangzhou, Guangdong China; 6 Department of Neurology, Guangzhou Women and Children’s Medical Center, Guangzhou Medical University, Guangzhou, Guangdong China; 7 Department of Endocrinology, The First Affiliated Hospital of Sun Yat-Sen University, Guangzhou, Guangdong China

**Keywords:** Physical activity (PA), Leisure sedentary behavior (LSB), COVID-19, Mendelian randomization (MR), Causal effect

## Abstract

**Background:**

The 2019 coronavirus disease pandemic (COVID-19) poses an enormous threat to public health worldwide, and the ensuing management of social isolation has greatly decreased opportunities for physical activity (PA) and increased opportunities for leisure sedentary behaviors (LSB). Given that both PA and LSB have been established as major influencing factors for obesity, diabetes and cardiometabolic syndrome, whether PA/LSB in turn affects the susceptibility to COVID-19 by disrupting metabolic homeostasis remains to be explored. In this study, we aimed to systematically evaluate the causal relationship between PA/LSB and COVID-19 susceptibility, hospitalization and severity using a Mendelian randomization study.

**Methods:**

Data were obtained from a large-scale PA dataset (N = 377,000), LSB dataset (N = 422,218) and COVID-19 Host Genetics Initiative (N = 2,586,691). The causal effects were estimated with inverse variance weighted, MR-Egger, weighted median and MR-PRESSO. Sensitivity analyses were implemented with Cochran’s Q test, MR-Egger intercept test, MR-PRESSO, leave-one-out analysis and the funnel plot. Risk factor analyses were further conducted to investigate the potential mediators.

**Results:**

Genetically predicted accelerometer-assessed PA decreased the risk for COVID-19 hospitalization (OR = 0.93, 95% CI 0.88–0.97; P = 0.002), while leisure television watching significantly increased the risk of COVID-19 hospitalization (OR = 1.55, 95% CI 1.29–1.88; P = 4.68 × 10^–6^) and disease severity (OR = 1.85, 95% CI 1.33–2.56; P = 0.0002) after Bonferroni correction. No causal effects of self-reported moderate to vigorous physical activity (MVPA), accelerometer fraction of accelerations > 425 milligravities, computer use or driving on COVID-19 progression were observed. Risk factor analyses indicated that the above causal associations might be mediated by several metabolic risk factors, including smoking, high body mass index, elevated serum triglyceride levels, insulin resistance and the occurrence of type 2 diabetes.

**Conclusion:**

Our findings supported a causal effect of accelerometer-assessed PA on the reduced risk of COVID-19 hospitalization as well as television watching on the increased risk of COVID-19 hospitalization and severity, which was potentially mediated by smoking, obesity and type 2 diabetes-related phenotypes. Particular attention should be given to reducing leisure sedentary behaviors and encouraging proper exercise during isolation and quarantine for COVID-19.

**Supplementary Information:**

The online version contains supplementary material available at 10.1186/s12967-022-03407-6.

## Introduction

Coronavirus disease 2019 (COVID-19), caused by severe acute respiratory syndrome coronavirus-2 (SARS-CoV-2), has struck humans globally with devastating impacts [1, 2]. The incredible rapid worldwide spread of COVID‐19 prompted the World Health Organization (WHO) to declare COVID‐19 a pandemic on 11 March 2020 [3, 4]. Since SARS-CoV-2 spreads from person to person among those in close contact (within two meters) primarily through respiratory droplets and subsequently causes COVID-19, controlling infectious sources, blocking the transmission routes, and protecting the susceptible population are still the main measures to control its transmission worldwide [5]. Therefore, considering the high contagiousness and serious adverse consequences of COVID-19, identifying behavior-related susceptibility factors of COVID-19 would facilitate effective policies and personalized treatments to control the epidemic spread and save social health care expenditures.

Due to mandatory quarantine requirements, the COVID-19 pandemic has created an environment that reduces habitual physical activity (PA) and promotes excessive time spent in leisure sedentary behaviors (LSB) [6]. Notably, a recent outbreak of highly contagious delta and omicron variants might further exacerbate this problem for a period of time [7]. Observational studies have reported a protective effect of PA on COVID-19 hospitalization [8]. LSB, defined as any awake behavior characterized by an energy expenditure of less than 1.5 metabolic equivalents in a sitting or reclining posture, such as sedentary television watching, computer using and driving [9, 10], have been shown to increase the risk of obesity [11], type 2 diabetes (T2D) [12], cardiovascular disease [13], cancers [14], and overall mortality [15], and to pose a substantial public health burden. Moreover, obesity [16, 17], type 2 diabetes [18, 19] and cardiometabolic disease [20, 21] are well-documented risk factors for severe COVID-19. Thus, we hypothesized that PA might decrease the risk of COVID-19 hospitalization and severity, whereas LSB potentially increases the risk by disrupting metabolic homeostasis. Indeed, recent (small-sample) studies have shown that people who engage in regular physical activity exhibit much lower odds of COVID-19 hospitalization, severe complications and mortality than those who are consistently inactive [22, 23]. However, direct evidence of the causal impact of LSB on COVID-19 susceptibility, severity and hospitalization characteristics remains lacking.

Owing to the inherent defects of conventional designs, existing observational studies are unable to entirely exclude the possibility of reverse causality and confounding factors, which potentially results in biased associations and conclusions [24]. Moreover, randomized controlled studies (RCTs) are unethical and impractical to perform on this topic due to the requirement of significant personnel resources and time-consuming follow-up. Mendelian randomization (MR) is increasingly applied to infer credible causal relationships between risk factors and disease outcomes [25]. Based on the random assortment of genetic variants during meiosis, MR used environmental exposure-related genetic variations as instrumental variables (IVs) to assess the association between exposures (e.g., LSB and PA) and outcomes (e.g., COVID-19 characteristics) [26]. Since genetic variants are randomly assigned at conception prior to disease onset, MR analysis could efficiently preclude confounding factors and identify causal determinants of a certain outcome [27].

Given the uncertainty about the causal association between LSB/PA and COVID-19, in the present study, we applied the MR design to evaluate the potential causal effect of PA/LSB on COVID-19 susceptibility, hospitalization and severity traits using large-scale genome-wide association study (GWAS) data. Overall, this study assesses the impacts of physical restrictions on COVID-19, identifies the susceptible subpopulations with a sedentary lifestyle during the pandemic, and provides constructive suggestions for preventive intervention strategies.

## Methods

### Study design

In this study, we performed a two-sample Mendelian randomization analysis to examine the causal effects of PA and LSB on COVID-19 susceptibility, hospitalization and severity using GWAS summary statistics. This instrumental-variable analysis mimics RCT with respect to the random allocation of single nucleotide polymorphisms (SNPs) in offspring (independent of confounding factors such as sex and age). In addition, this MR design has to fulfill three assumptions: (i) genetic instruments predict the exposure of interest (P < 5 × 10^–8^); (ii) genetic instruments are independent of potential confounders; (iii) genetic instruments affects the outcome only via the risk factors [28].

### Exposure GWAS-physical activity and leisure sedentary behaviors

GWAS data for physical activity were obtained from the UK Biobank [29, 30]. The data were adjusted for covariates including age, sex, genotyping chip, first ten genomic principal components, center, season (month) for center visit or wearing an accelerometer. Physical activity included three phenotypes: self-reported moderate to vigorous physical activity (MVPA), accelerometer-assessed PA, and accelerometer-assessed fraction accelerations > 425 milligravities. A detailed description of the above PA phenotypes can be found in the original article of Doherty Aiden et al. [29]. The IVs for PA were identified by the following criteria raised by Martin Bahls et al.: (1) SNPs at the genome-wide significance level (P < 5 × 10^–8^); (2) SNP clumping using the PLINK algorithm (r^2^ threshold = 0.001 and window size = 10 mB); and (3) removal of SNPs exhibiting potential pleiotropic effects [31]. One of the SNPs for MVPA was missed in the GWAS of COVID-19. Therefore, 16, 7 and 7 SNPs were used as IV for MVPA, accelerometer measure PA and fraction accelerations > 425 milligravities in our study, respectively (Additional file 1: Table S1–S3).

Candidate genetic instruments for leisure sedentary behaviors were extracted from the latest summary-level GWAS consisting of 422,218 European ancestry participants from the UK Biobank [9]. In the present GWAS meta-analyses, leisure sedentary behaviors mainly included three categories: leisure television watching, leisure computer use, and driving. The amount of time participants spent on each of the three behaviors was recorded by answers to the following questions: “On a typical day, how many hours do you spend on watching television?”, “On a typical day, how many hours do you spend using a computer? (Do not include using a computer at work)” and “On a typical day, how many hours do you spend driving?”. The reported average daily time spent watching television watching was 2.8 hours (h) [standard deviation (SD) 1.5 h], computer use was 1.0 h (SD 1.2 h), and driving was 0.9 h (SD 1.0 h). After adjusting covariates, including age, sex, body mass index, smoking status, hypertension, diabetes, Townsend deprivation index, physical activity levels, alcohol use per week and years of education, 209 television watching-related SNPs, 35 computer use-related SNPs, and 4 driving-related SNPs were identified (P < 1 × 10^–8^) [9].

In addition, rigorous filtering steps were performed to control the SNP quality before MR analysis. First, we aggregated SNPs in linkage disequilibrium (LD, R^2^ ≥ 0.001 and within 10 mb). Second, to quantify the strength of the genetic instruments, SNPs with an F statistic less than 10 were excluded. Third, SNPs were associated with the appropriate exposure at the genome-wide significance threshold P < 5 × 10^−8^. Fourth, harmonizing processes were conducted to exclude ambiguous and palindromic SNPs (EAF > 0.42). Finally, SNPs with potential pleiotropy were removed after MR-pleiotropy residual sum and outlier (MR-PRESSO), and MR analysis was reperformed to evaluate the robustness. Finally, 84, 21 and 4 SNPs were used as IVs respectively for television watching, computer use and driving, respectively. Details of the SNPs used as instrumental variables were displayed in Additional file 1:Tables S4–S6.

### Outcomes in GWAS: COVID-19 phenotypes

Summary statistics for COVID-19 phenotypes were extracted from the latest version of the COVID-19 Host Genetics Initiative (HGI) GWAS meta-analyses (Round 6, June 2021) [32]. In total, 2,586,691 participants of European ancestry were enrolled. COVID-19 phenotypes included COVID-19 susceptibility, hospitalization, and severe clinical outcomes. Briefly, the diagnosis of COVID-19 cases was based on laboratory-confirmed infection of severe acute respiratory syndrome coronavirus 2 (SARS-CoV-2), electronic health records or doctor diagnosis of COVID-19, or self-reported COVID-19 infections from the patients. The outcome of COVID-19 susceptibility compared COVID-19 cases (N = 112,612) with population controls without a history of COVID-19 (N = 2,474,079). Hospitalized COVID-19 cases were defined as having laboratory-confirmed SARS-CoV-2 infection or being hospitalized with COVID-19-related symptoms. The outcome of COVID-19 hospitalization compared hospitalized COVID-19 cases (N = 24,274) with population controls not experiencing hospitalization for COVID-19 (including those without COVID-19) (N = 2,061,529). Severe COVID-19 cases were defined as hospitalized COVID-19 individuals requiring respiratory support, including intubation, continuous positive airway pressure (CPAP), bilevel positive airway pressure (BiPAP), continuous external negative pressure, or high-flow nasal cannula. The outcome of COVID-19 severity compared severe COVID-19 cases (N = 8,779) with individuals without severe COVID-19 (including those without COVID-19) (N = 1,001,875). The COVID-19 GWAS dataset was adjusted for age, age^2^, sex, age × sex, principal components and study-specific covariates by the original GWAS investigators [33].

### Mendelian randomization analyses

To address the potential pleiotropic effects of genetic variants, four MR analytical methods were performed to evaluate the causal effects of LSB and PA on COVID-19 in this study. We applied standard inverse variance weighted (IVW) estimates for the main analysis, which combined the Wald ratio of each SNP on the outcome and obtained a pooled causal estimate. Overdispersion was allowed in this method. Furthermore, additional MR analyses, such as MR-Egger regression, weighted median, and Mendelian randomization pleiotropy residual sum and outlier (MR-PRESSO) methods, were implemented as complements to the IVW, because these methods could provide more robust estimates over a wider range of scenarios. MR-Egger regression could provide a test for unbalanced pleiotropy and considerable heterogeneity, whereas it requires a larger sample size for the same underexposure variation [34]. The weighted median method provides consistent estimates of effect, when at least half of the weighted variance provided by horizontal pleiotropy is valid [35]. Moreover, similar to previous studies by our group as well as other researchers, a stringent instrument P value threshold was used and recalculated if the results from different MR analyses were inconsistent [36].

### Sensitivity analysis

Horizontal pleiotropy occurs when genetic variants associated with the exposure of interest (LSB) directly affect the outcome (severe COVID-19) through multiple pathways other than the hypothesized exposure. Therefore, we further conducted Cochran’s Q statistic, funnel pot, leave-one-out (LOO) analyses and MR-Egger intercept tests to detect the presence of pleiotropy and assess the robustness of the results. Specifically, heterogeneity was detected if the P value of the Cochran Q test was less than 0.05. We also appraised horizontal pleiotropy based on the intercept term derived from MR-Egger regression. To determine whether the causal estimate was driven by any single SNP, we performed LOO analysis, through which each exposure-associated SNP was discarded by turns to repeat the IVW analysis.

### Risk factors

To explore the underlying mechanisms genetically linking PA and LSB with COVID-19, we further calculated several potential mediators for analysis, including body mass index (BMI), serum lipid levels, type 2 diabetes, fasting insulin level, and smoking. Genetic effects on the BMI were obtained from the Genetic Investigation of Anthropometric Traits (GIANT) consortium [37]. For the lipid profile, we used GWAS data on triglycerides and total cholesterol from the UK Biobank [38]. Genetic information for T2D was obtained from the Diabetes Genetics Replication and Meta-analysis (DIAGRAM) consortium [39]. For fasting insulin, we obtained the GWAS data from the Meta-Analyses of Glucose and Insulin-related traits Consortium (MAGIC) [40]. GWAS summary data for two tobacco smoking phenotypes were obtained from the Tobacco and Genetics (TAG) consortium, including former versus current smokers and the number of cigarettes smoked per day [41]. Detailed information on each data source was displayed in Table 1. PA and LSB were treated as exposures, and the above potential risk factors were used as outcomes to perform Mendelian randomization. Estimates of IVW were assessed as the main results. P < 0.05 was regarded as significant.

**Table 1:** Data source of the COVID-19-related risk factors

Traits	Consortium	Sample size	Ancestry	PubMed ID
Body mass index	GIANT	322,154	European	25673413
Total cholesterol	UK Biobank	17,802	European	31217584
Triglycerides	UK Biobank	441,016	European	32203549
Type 2 diabetes	DIAGRAM	69,033	European	22885922
Fasting insulin	MAGIC	51,750	European	22581228
Former versus current smoker	TAG	41,969	European	20418890
Cigarettes smoked per day	TAG	68,028	European	20418890

### Statistical analysis

To account for multiple testing in our primary analyses, a Bonferroni-corrected threshold of P < 0.0028 (α = 0.05/18 outcomes) was applied. The MR estimates were presented as odds ratios (OR) with corresponding 95% confidence intervals (CI), which provided an estimate of the relative COVID-19 risk caused by each standard deviation (SD) increase in the time spent on television watching, computer use and driving. All analyses were performed by the packages TwoSampleMR (version 0.4.25) and MRPRESSO (version 1.0) in R (version 3.6.1).

## Results

### MR estimates

**Fig. 1:**
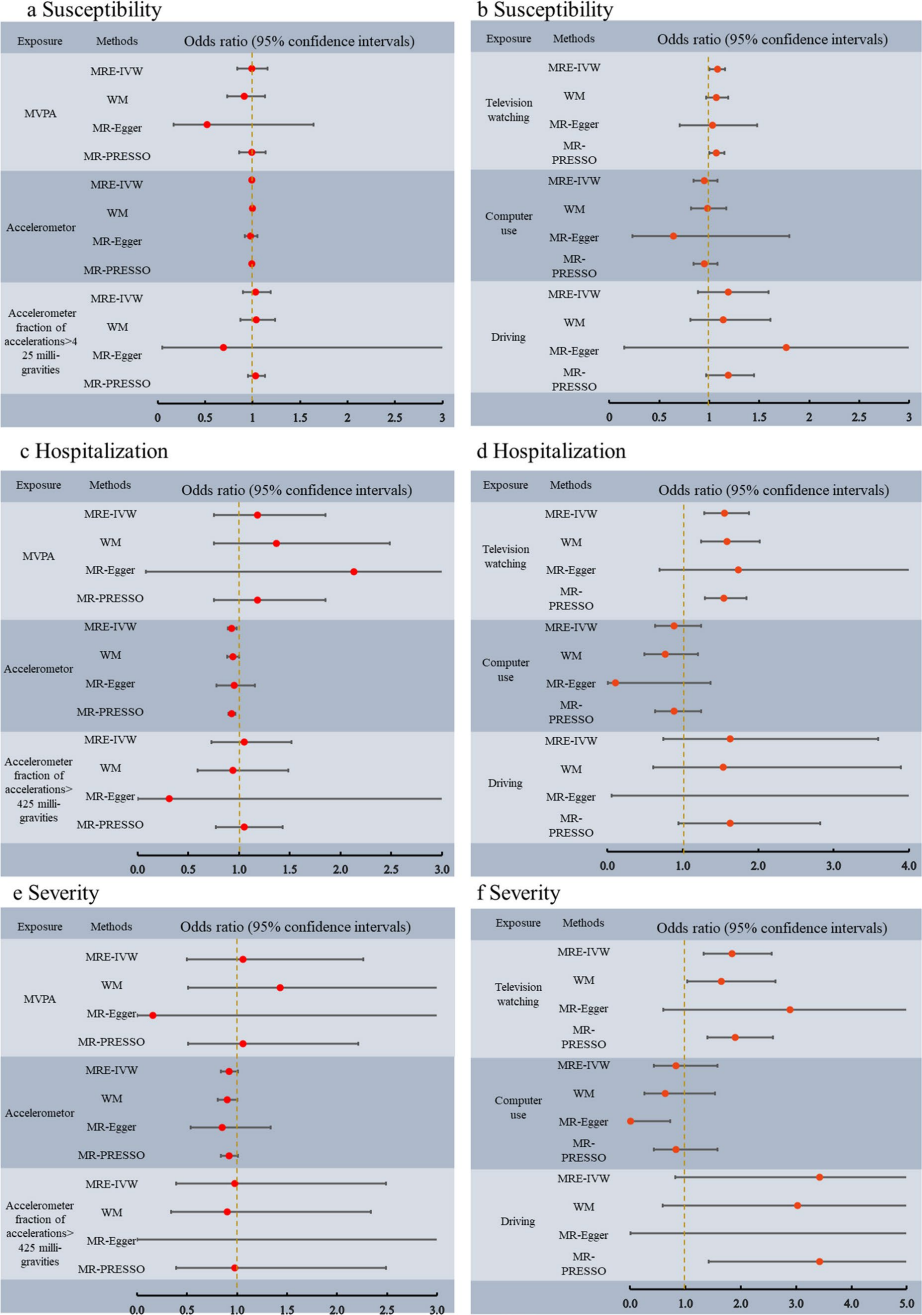
Causal effects for physical activity and leisure sedentary behaviors on COVID-19 susceptibility (**a, b**), hospitalization (**c, d**) and severity (**e, f**). Summary of the Mendelian randomization (MR) estimates derived from the inverse-variance weighted (IVW), weighted median (WM), MR-Egger, and MR-PRESSO methods. MVPA: self-reported moderate to vigorous physical activity

Among the tested PA phenotypes, IVW analysis indicated that accelerometer assessed PA decreased the risk for COVID-19 hospitalization (OR = 0.93, 95% CI 0.88–0.97; P = 0.002). The results from other MR methods showed a consistent but nonsignificant direction (Additional file 1: Table S7 and Fig. 1c). Genetically proxied leisure television watching was significantly associated with an increased risk of COVID-19 hospitalization (OR = 1.55, 95% CI 1.29–1.88; P = 4.68 × 10^–6^), and greater disease severity (OR = 1.85, 95% CI 1.33–2.56; P = 0.0002) in the IVW analysis (Fig. 1d–e). Similar causal estimates for COVID-19 hospitalization were obtained from other MR models, including WM (OR = 1.58, 95% CI 1.24–2.02; P = 0.0002), and MR-PRESSO regression (OR = 1.54, 95% CI 1.29–1.84; P = 7.81 × 10^–6^). Moreover, a positive causality between genetic predisposition toward leisure television watching and COVID-19 severity was also detected using MR-PRESSO analysis (OR = 1.90, 95% CI 1.39–2.58; P = 0.0001), whereas WM analysis presented a nominally significant result (OR = 1.65, 95% CI 1.04–2.62; P = 0.03) (Fig. 1f). However, we did not observe evidence of causal association between television watching and COVID-19 susceptibility, hospitalization or severity using the MR-Egger analysis. In addition, no causal association was detected between genetic liability for MVPA, accelerometer fraction of accelerations > 425 milligravities, computer use or driving and COVID-19 risk (Fig. 1a–f).

### Sensitivity analyses

**Fig. 2:**
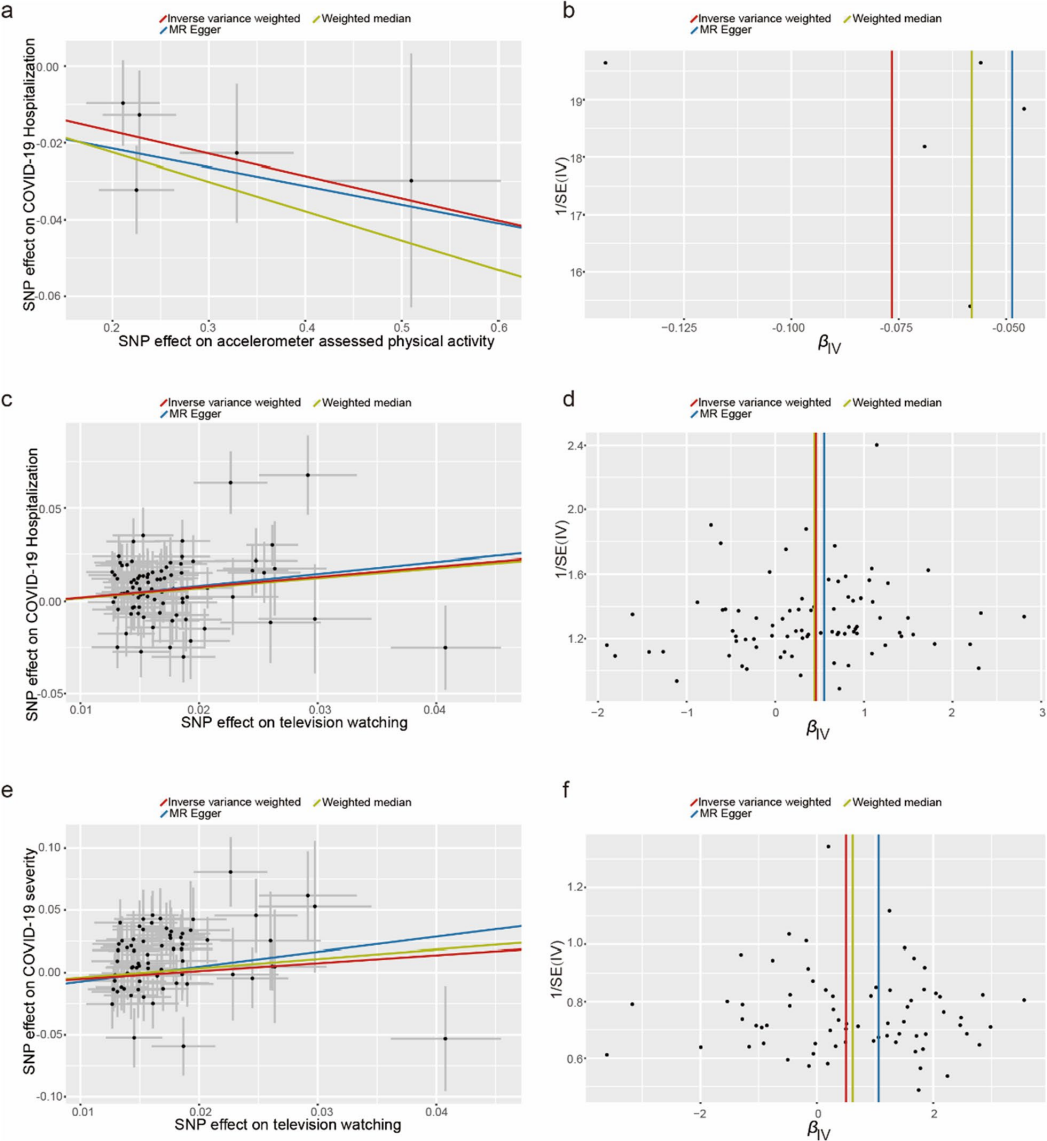
Scatter plots and funnel plots from genetically predicted accelerometer assessed physical activity and leisure watching TV on COVID-19 hospitalization and severity. **a**, **b** genetically predicted accelerometer assessed physical activity on COVID-19 hospitalization; **c**, **d** genetically predicted television watching on COVID-19 hospitalization; **e, f** genetically predicted television watching on COVID-19 severity

To assess the robustness of the above results, a series of sensitivity analyses, including Cochran’s Q test, MR-Egger intercept test, and MR-PRESSO global test, were conducted (Table 2). All P values of the MR-Egger intercept tests were > 0.05, indicating that no horizontal pleiotropy existed. However, heterogeneity was observed in the Q test analysis between accelerometer-assessed PA and COVID-19 hospitalization (Q = 17.52, P = 0.008), television watching and COVID-19 hospitalization (Q = 112.82, P = 0.01), television watching and COVID-19 severity (Q = 90.33, P = 0.04), and driving and COVID-19 susceptibility (Q = 116.22, P = 0.01). Even though heterogeneity was detected in certain results, it did not invalidate the MR estimates as random-effect IVW in the current study, which might balance the pooled heterogeneity. In addition, Egger intercepts did not detect any pleiotropy, suggesting that no pleiotropic bias was introduced to MR estimates in the context of heterogeneity (Fig. 2a, c, e). No heterogeneity was detected in other analyses. Moreover, LOO analysis revealed that no SNP drove the results, and funnel plots were symmetrical (Fig. 2b, d, f), indicating that none of the estimates were violated (Additional file 2: Figures S1–S2).

**Table 2:** Sensitivity analysis of the causal association between leisure sedentary behaviors and the risk of COVID-19

Exposure	Outcome	Cochran Q test		MR-Egger		MR-PRESSO P value
		Q value	*P*	Intercept	*P*	
MVPA	Susceptibility	5.37	0.50	0.009	0.29	0.70
	Hospitalization	17.08	0.31	− 0.008	0.73	0.33
	Severity	13.98	0.53	0.03	0.48	0.54
Accelerometer assessed PA	Susceptibility	11.77	0.70	0.002	0.86	0.52
	Hospitalization	17.52	0.008	0.02	0.59	0.69
	Severity	8.70	0.19	0.02	0.72	0.24
Accelerometer fraction of accelerations > 425 milligravities	Susceptibility	2.44	0.87	0.01	0.77	0.88
	Hospitalization	4.20	0.65	0.03	0.74	0.68
	Severity	11.86	0.07	− 0.07	0.77	0.08
Television watching	Susceptibility	78.90	0.12	0.0009	0.78	0.23
	Hospitalization	112.82	0.01	− 0.002	0.81	0.03
	Severity	90.33	0.04	− 0.002	0.88	0.02
Computer use	Susceptibility	21.95	0.34	0.006	0.46	0.35
	Hospitalization	21.63	0.36	0.03	0.12	0.38
	Severity	22.55	0.26	0.08	0.06	0.25
Driving	Susceptibility	116.22	0.01	− 0.006	0.78	0.70
	Hospitalization	1.45	0.69	− 0.05	0.43	0.69
	Severity	1.13	0.77	− 0.09	0.44	0.77

MVPA: self-reported moderate to vigorous physical activity

### Risk factor analysis

To further investigate the potential mediators linking television watching to an increased risk of COVID-19, we assessed its effects on several common risk factors for COVID-19 using MR methods. As shown in Table 3, the causal effect of accelerometer assessed PA on hospitalization was not biased by potential risk factors. For LSB, every 1 SD hour (2.8 h) increase in watching television was found to be significantly associated with 0.66 mmol/L higher total cholesterol, 31% lower odds of smoking cessation among smokers, 0.25 higher BMI, 0.26 mmol/L higher serum triglycerides, 82% higher risk of T2D, and 0.15 pmol/L higher fasting insulin. Collectively, our findings suggested that metabolic disorders and smoking behaviors might be responsible for LSB-linked COVID-19 susceptibility according to the risk factor analyses.

**Table 3:** Risk factors analysis

Exposure	Outcomes	IVW		Heterogeneity		MR-Egger method	
		Causal effect (95% CI)	*P*	Q value	*P*	Intercept	*P*
Accelerometer assessed physical activity	Body mass index	− 0.01 (− 0.05,0.025)	0.56	16.91		− 0.019	
Accelerometer assessed physical activity	Total cholesterol	− 0.82 (− 2.52,0.88) mmol/L	0.34	4.43	0.49	0.23	0.84
Accelerometer assessed physical activity	Triglycerides	− 0.008 (− 0.27, 0.01) mmol/L	0.42	43.52	< 0.001	− 0.008	0.50
Accelerometer assessed physical activity	Type 2 diabetes	1.04 (0.93, 1.16)	0.49	2.35	0.31	− 0.1	0.39
Accelerometer assessed physical activity	Fasting insulin	1.01 (0.99, 1.04)		3.99		− 0.014	
Accelerometer assessed physical activity	Former versus current smoker	0.97 (0.86, 1.08)	0.56	10.13	0.04	0.13	0.19
Accelerometer assessed physical activity	Cigarettes smoked per day	1.12 (0.77, 1.61)	0.56	0.80	0.94	0.08	0.80
Television watching	Body mass index	0.25 (0.15, 0.35)	1.53 × 10^–7*^	148.43	< 0.001	− 0.0003	0.95
Television watching	Total cholesterol	0.66 (− 5.84, 7.18) mmol/L	0.84	131.1	0.001	0.23	0.40
Television watching	Triglycerides	0.26 (0.17, 0.35) mmol/L	1.22 × 10^–8 *^	844.7	< 0.001	0.003	0.30
Television watching	Type 2 diabetes	1.82 (1.25, 2.65)	1.79 × 10^–3 *^	95.54	0.0014	− 0.007	0.71
Television watching	Fasting insulin	0.15 (0.09, 0.21) pmol/L	2.52 × 10^–6 *^	85.87	0.02	0.004	0.23
Television watching	Former versus current smoker	0.69 (0.53, 0.90)	0.007 ^*^	77.57	0.22	− 0.006	0.62
Television watching	Cigarettes smoked per day	0.73 (− 0.82, 2.28)	0.36	95.85	0.02	0.04	0.63

^*^P < 0.05

## Discussion

Using large-scale GWAS data from the UK Biobank and the COVID-19 Host Genetics Initiative (HGI), this study implemented multiple MR approaches to appraise the possible causal association of PA/LSB with COVID-19 susceptibility and progression. We demonstrated that accelerometer-assessed PA causally decreased the risk for COVID-19 hospitalization (7%), and leisure television watching causally increased the rate of COVID-19 hospitalization (55%) and severity (85%). In view of the worldwide prevalence of COVID-19, the emergence of new variants and the necessity of isolation measures, our study provides novel insight into reducing the risk of COVID-19 hospitalization and progression by underscoring the necessity of physical activity management during quarantine and stay-at-home mandates.

With the COVID-19 pandemic, people were prone to reduce PA and increase time spent in LSB, due to social distancing restrictions and isolation requirements [22]. However, COVID-19 is a novel and rapidly changing disease caused by SARS-CoV-2 and its variants, the related risk factors for which are even more unclear. Notably, sustained physical inactivity and sedentary behaviors are typically associated with poor physical and mental health and an increased risk of multiple chronic diseases, including obesity, type 2 diabetes, cardiovascular disorders and cancer [11, 12, 14, 15]. A Community-Based Cohort Study in the UK demonstrated that unhealthy behaviors accounted for 51% of the population attributable fraction of severe COVID-19, according to the estimates of risk factor prevalence [23]. Specifically, unhealthy lifestyles of physical inactivity, smoking and obesity were demonstrated to increase the risk of COVID-19 with ORs varying from 1.32 to 2.05 [23]. Despite of these results from small-scale observational studies, direct evidence for the causal relationship between sedentary behaviors and COVID-19 is still lacking [42]. Compared with relatively impractical large-scale prospective clinical trials requiring long-term observation, the MR study sheds a new light on the causal relationship between PA/LSB and COVID in a time- and cost-efficient way.

Intriguingly, leisure television watching causally increased COVID-19 hospitalization and severity rate, which was in accordance with multiple investigations that recognized television watching as the main leisure-time associated sedentary behavior [43]. This result implicated a biological heterogeneity behind domain-specific sedentary behaviors and indicated the importance of distinguishing different sedentary behaviors during COVID-19 management. Compared with other sedentary traits, television watching was the most canonical leisure sedentary behavior that shifted the energy balance toward an energy surplus through fewer breaks, lower energy expenditure and excessive energy (especially snacks) intake [9, 44, 45]. Recent studies illustrated that low energy expenditure manifested by increased BMI was associated with a higher risk of COVID-19 hospitalization and severe complications, including sepsis and respiratory failure [46]. Consistent with these previous findings, our MR analyses clearly indicated a causal association of television watching with COVID-19 hospitalization and severity but not COVID-19 susceptibility. Our results suggested that particular attention should be given to the lifestyle of COVID-19 patients during quarantine. For example, patients should be encouraged to reduce time spent watching television and appropriately increase energy-consuming exercise to improve physical fitness.

Additionally, our in-depth analyses elicited several potential explanations for this causal association between television watching and COVID-19. We first identified several obesity-related phenotypes, including BMI, triglycerides, fasting insulin and T2D, that might play a mediating role linking television watching with COVID-19 incidence. Substantial evidence suggests that obesity and T2D can lead to chronic low-grade inflammation and immune dysregulation, resulting in compromised activation and function of these adaptive immune cells in response to SARS-CoV-2 [47]. Zhang et al. reported that elevated total cholesterol and ApoB levels might increase the risk of COVID-19 infection using MR analyses. Similar to these findings, our results further implied that elevated triglyceride levels should also be monitored in COVID-19 patients [48]. In addition, previous research showed that the proportion of current smokers accumulated with the increase in time spent watching television [45]. Likewise, our study confirmed that increased television watching led to a lower likelihood of smoking cessation among smokers. Given that smoking behavior is a well-established cause of COVID-19, it might serve as a key intermediate factor in the television watching-COVID-19 pathway. Additionally, previous MR studies unveiled a causal association between psychiatric disorders and COVID-19 [49, 50]. Compared with computer use and driving, television watching is a more immersive, less reflective and communicative form of leisure entertainment. Sustained television viewing was consistently accompanied by poor physical and mental health (such as anxiety and depression), which might partially contribute to COVID-19 as well. Finally, elderly people tended to spend more time on watching television as a leisure entertainment, whereas the young population were more likely to use computers or drive. Thus, more attention should be given to elderly people with respect to their physical activity instructions during COVID-19 pandemic social restrictions. Notably, further studies are warranted to determine the exact degree of LSB mediation for COVID-19. Without specific mediation analysis, the direct effect of television watching on COVID-19 could not be determined.

Taken together, our findings supported the hypothesis that LSB (especially television viewing) increases COVID-19 risk, and strategies to enhance physical activity during the COVID-19 pandemic deserve particular attention. First, during the quarantine period for controlling infection sources and cutting off the transmission, residents should be encouraged to appropriately increase their energy consumption behaviors rather than engage in leisure sedentary behaviors. Light-to-moderate-intensity activities, such as housework and brisk walking, should also be encouraged [51]. Moreover, anti-smoking advocacy should be routinely performed among COVID-19 patients, which could be of great significance in delaying the progression of the disease, reduce its severity, and promote early recovery. In addition, as a high-risk group for COVID-19 and major audience for television, individuals with obesity or type 2 diabetes, especially elderly adults, should be assisted in reducing leisure television viewing time and participating in physical activities.

In general, the statistical power of the IVW approach is dramatically higher than that of the other MR approaches, especially MR-Egger [52]. Confidence intervals were calculated from the same equations that generated P values. Therefore, not surprisingly, the MR–Egger results with low statistical power had wider confidence and nonsignificant P values when compared with IVW in the present study. For the same reason, IVW was usually used as the main method to screen for potentially significant results. Sensitivity analyses and other MR methods were implemented to ensure the robustness of IVW estimates. IVW estimates would be biased if horizontal pleiotropy existed. In this scenario, the MR-Egger estimates should be referenced because this method adapts the IVW analysis by allowing the horizontal pleiotropic effect across all SNPs to be unbalanced or directional [53]. In most MR analyses, researchers have strengthened the requirement for consistent beta direction in all MR approaches, which was also used in our study [54, 55].

However, several limitations should be taken into account in our study. First, since this study was conducted among European ancestry participants, the results cannot be immediately generalized to other ethnic groups with different lifestyles and cultural backgrounds. Second, since MR analyses extrapolated causal hypotheses by exploiting the random allocation of genetic variants, it was difficult to completely distinguish between mediation and pleiotropy using MR approaches. The generous variants in our genome probably affect one or more phenotypes. Third, there was a lack of additional mediator analysis and observation studies to further confirm the metabolic mechanisms involved in the causal relationship between television watching and COVID-19. Due to the limitations of the UK Biobank data, future studies are required to confirm the causality and explore potential mechanisms, which is indispensable for developing relevant clinical recommendations.

In conclusion, leveraging large-scale genetic summary data, our study first strengthened the evidence that PA/LSB was causally associated with the hospitalization and severity of COVID-19. Further work is warranted to decipher the underlying mechanisms linking leisure television watching with COVID-19. Given the COVID-19 pandemic and the necessity of social quarantine, intensive attention should be given to lifestyle management, such as reducing leisure sedentary behaviors and encouraging proper exercise, to combat COVID-19.

## Supplementary Information

**Additional file 1: Supplementary tables.  Table S1.** Instrument variables of MVPA. **Table S2.** Instrument variables of accelerations assessed physical activity. **Table S3.** Instrument variables of fraction accelerations > 425 milli-gravities. **Table S4.** Instrument variables of television watching. **Table S5.** Instrument variables of computer used. **Table S6.** Instrument variables of driving. **Table S7.**MR estimates of the causal association between physical activity and leisure sedentary behaviors and the risk of COVID-19.

**Additional file 2: Supplementary figures. Figure S1.** Forest plot (**a**) and leave-one-out analysis (**b**) for accelerometer assessed physical activity on COVID-19 hospitalization. **Figure S2**. Forest plot (**a**) and leave-one-out analysis (**b**) for leisure television watching on COVID-19 hospitalization. **Figure S3**. Forest plot (**a**) and leave-one-out analysis (**b**) for leisure television watching on COVID-19 severity.
