# Supplementary material for: Causal relationship between physical activity, leisure sedentary behaviors and COVID-19 risk: a Mendelian randomization study
Source: J Transl Med. 2022 May 13;20:216. doi: 10.1186/s12967-022-03407-6 (PMC9100292; doi:10.1186/s12967-022-03407-6)
Supplement: Supplementary file 2 — Additional file 2: Supplementary figures. Figure S1. Forest plot (a) and leave-one-out analysis (b) for accelerometer assessed physical activity on COVID-19 hospitalization. Figure S2. Forest plot (a) and leave-one-out analysis (b) for leisure television watching on COVID-19 hospitalization. Figure S3. Forest plot (a) and leave-one-out analysis (b) for leisure television watching on COVID-19 severity. [file 12967_2022_3407_MOESM2_ESM.docx]

**Causal Relationship Between Physical Activity, Leisure Sedentary Behaviors and COVID-19 Risk: A Mendelian Randomization Study**

Xiong Chen,MD ^1,2,*^, Xiaosi Hong, MM ^3,*^, Wenjing Gao, MM ^4^, Shulu Luo, MM ^5^, Jiahao Cai, MD ^6^,^†^, Guochang Liu, PhD ^1,2, †^, Yinong Huang, MD ^7, †^

Additional file 2: figures


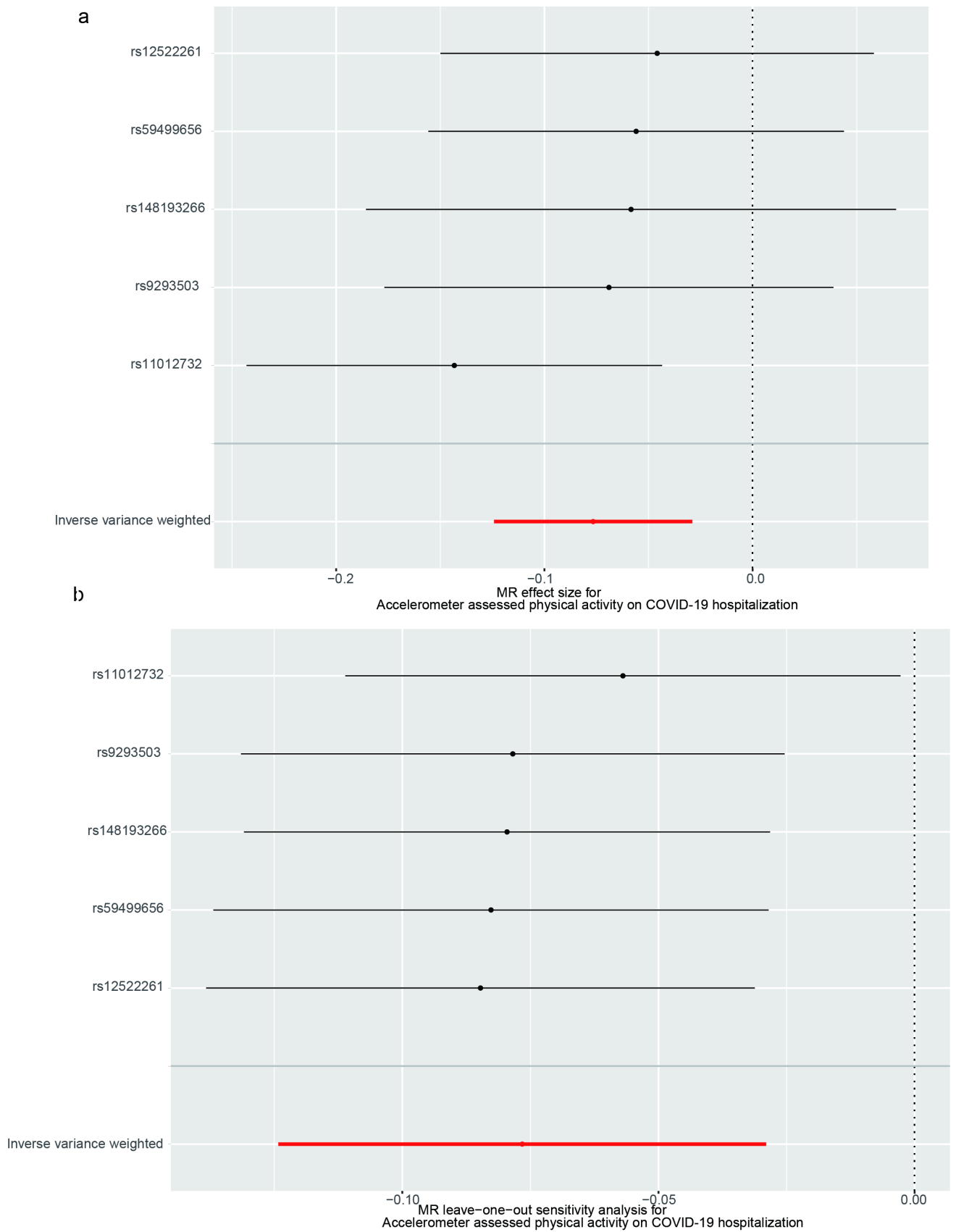


Figure S1. forest plot(a) and leave-one-out analysis(b) for accelerometer assessed physical activity on COVID-19 hospitalization.


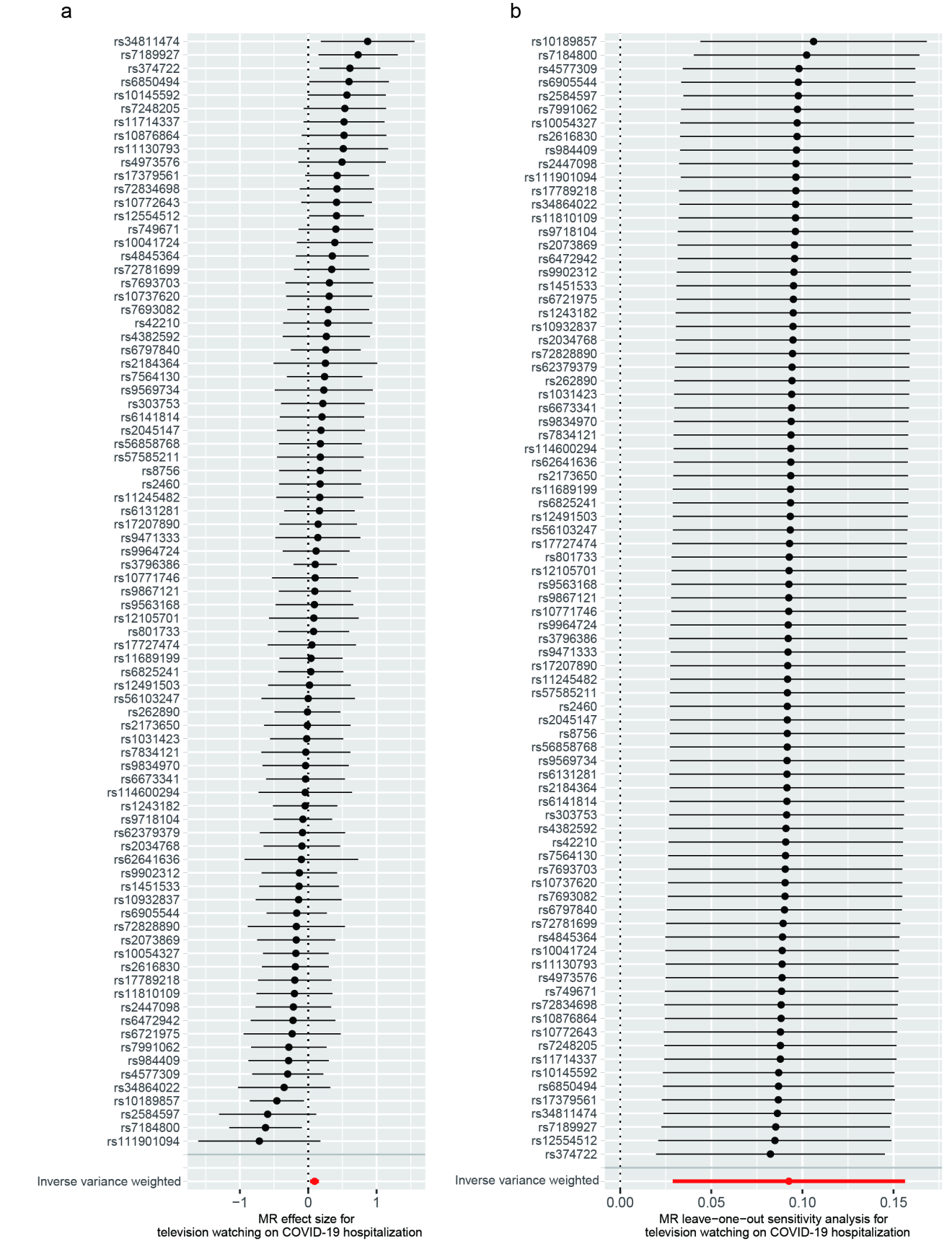


Figure S2. forest plot(a) and leave-one-out analysis(b) for leisure television watching on COVID-19 hospitalization.


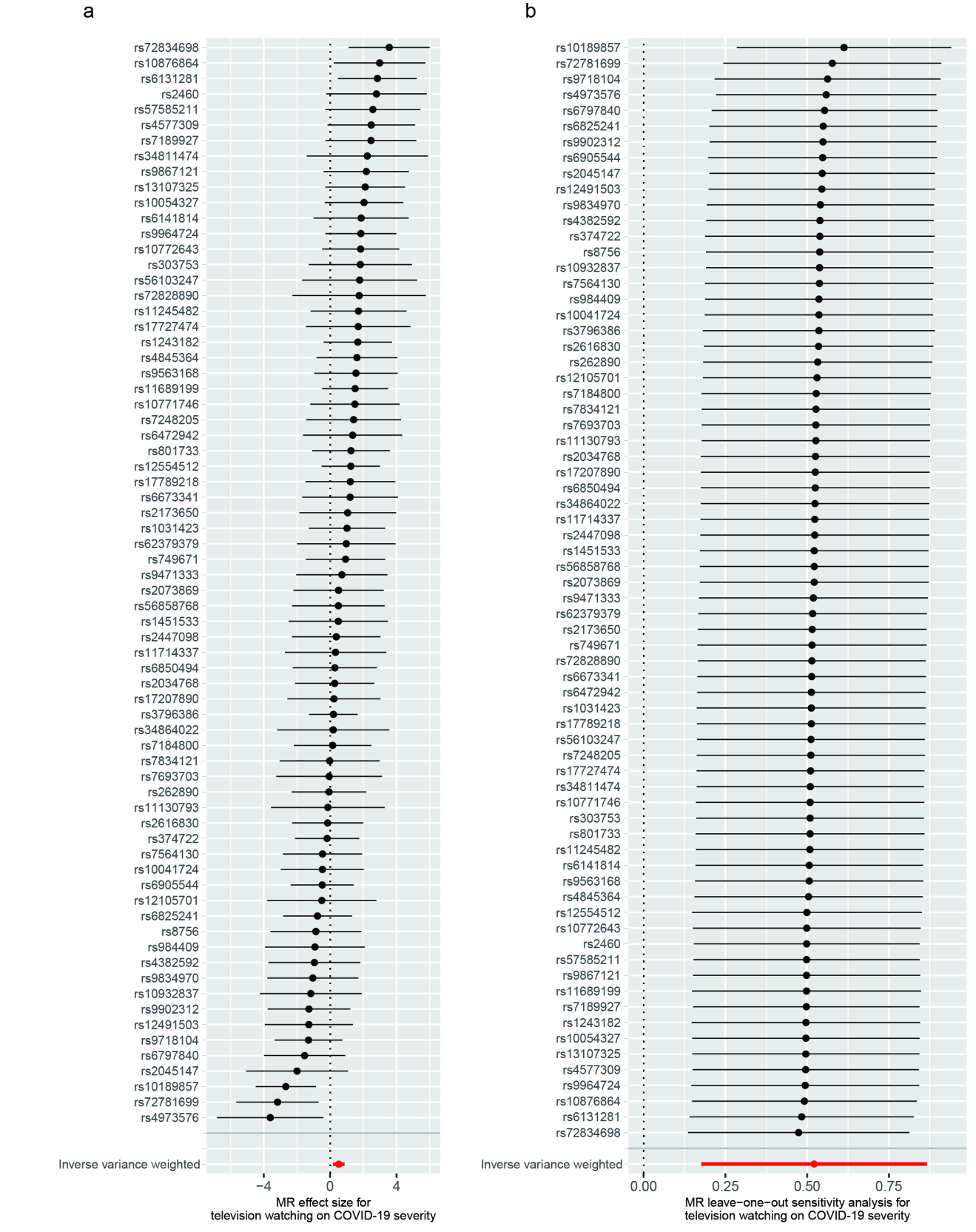


Figure S3. forest plot(a) and leave-one-out analysis(b) for leisure television watching on COVID-19 severity.

**Additional file 2: code for MR analysis**

library(TwoSampleMR)

GRS_file <- system.file("extdata/PA.csv", package="TwoSampleMR")

GRS_exp_dat <- read_exposure_data( filename = GRS_file, sep = ",", snp_col = "SNP",beta_col = "beta",

se_col = "se",effect_allele_col = "effect_allele",other_allele_col = "other_allele", eaf_col = "eaf", pval_col = "P_value", phenotype_col = "exposure")

outcome_dat <- read_outcome_data(snps = GRS_exp_dat$SNP,filename = "PA_Severe_population.csv",sep = ",",snp_col = "SNP",beta_col = "beta",se_col = "se",effect_allele_col = "effect_allele",other_allele_col = "other_allele", eaf_col = "eaf", pval_col = "P_value")

dat <- harmonise_data(exposure_dat = GRS_exp_dat, outcome_dat = outcome_dat)

write.csv(dat,"PA_Hospital_population_dat.csv")

res <- mr(dat)

res

write.csv(res,"covid_population_LSB_dat1.2_res.csv")

h <- mr_heterogeneity(dat)

write.csv(h,"covid_population_LSB_dat1.2_h.csv")

p <- mr_pleiotropy_test(dat)

write.csv(p,"covid_population_LSB_dat1.2_p.csv")

res_single <- mr_singlesnp(dat)

res_loo <- mr_leaveoneout(dat)

res <- mr(dat, method_list=c("mr_egger_regression", "mr_ivw","mr_weighted_median"))

p1 <- mr_scatter_plot(res, dat)

p1[[1]]

res_single <-mr_singlesnp(

dat,

parameters = default_parameters(),

single_method = "mr_wald_ratio",

all_method = "mr_ivw")

))

p2 <- mr_forest_plot(res_single)

p2[[1]]

res_loo <- mr_leaveoneout(dat)

p3 <- mr_leaveoneout_plot(res_loo)

p3[[1]]

p4 <- mr_funnel_plot(res_single)

p4[[1]]

#MR-PRESSO

library(MRPRESSO)

mr_presso(BetaOutcome = "beta.outcome", BetaExposure = "beta.exposure", SdOutcome = "se.outcome", SdExposure = "se.exposure", OUTLIERtest = TRUE, DISTORTIONtest = TRUE, data = dat, NbDistribution = 10000, SignifThreshold = 0.05,)
